# Identification Checks and Underage Sales of Tobacco Products in New Jersey, 2019-2022

**DOI:** 10.1001/jamanetworkopen.2024.57319

**Published:** 2025-01-30

**Authors:** Amanda Y. Kong, Mary Hrywna, Christopher Ackerman, Joseph G. L. Lee, Cristine D. Delnevo

**Affiliations:** 1 Department of Social Sciences and Health Policy, Division of Public Health Sciences, Wake Forest University School of Medicine, Winston-Salem, North Carolina; 2 Rutgers Institute for Nicotine & Tobacco Studies, Rutgers University, New Brunswick, New Jersey; 3 Department of Health Behavior, Society & Policy, Rutgers School of Public Health, Piscataway, New Jersey; 4 Department of Health Education and Promotion, College of Health and Human Performance, East Carolina University, Greenville, North Carolina

## Abstract

This cross-sectional study examines compliance with Tobacco 21 laws in New Jersey between 2019 and 2022.

## Introduction

In 2017, New Jersey became one of the first states to pass legislation to increase the minimum legal sales age of tobacco from 18 to 21 years (or Tobacco 21, hereafter T21), followed by later federal T21 enactment in 2019. This study examines factors associated with T21 compliance in a 3-wave longitudinal sample of New Jersey tobacco retailers between 2019 and 2022.

## Methods

The Rutgers institutional review board determined this cross-sectional study was non–human participants research; thus, informed consent was not needed, in accordance with 45 CFR §46.^1,2^ This study followed the STROBE reporting guidelines. Methods are detailed in the eMethods in Supplement 1: in brief, we randomly sampled tobacco retailers in high and low population density municipalities within a 25-mile radius of New Brunswick, New Jersey. We calculated tobacco retailer availability (TRA) by geocoding retailer addresses and determined the total number of tobacco retailers per square mile within each census tract. We fit generalized estimating equation models to account for nesting of visits within retailers and included a year fixed effect. Models tested associations with 95% CIs between product type, store type, and TRA with 3 dichotomous primary outcome variables: identification (ID) checked, ID scanned, and sale completed.

## Results

Underage buyers (aged 18-20 years) completed 2663 purchase attempts for tobacco products in 70 unique retailers between August 2019 and September 2022. Nearly 40% of purchase attempts were made in nonchain convenience stores, followed by chain convenience stores (33.2%), other store types (11.5%), drug stores (8.8%), and gas kiosks (6.7%). Cigarettes (32.7%) and cigars and/or cigarillos (32.3%) were the most common products attempted for purchase, followed by electronic cigarettes (24.5%) and nicotine pouches (10.5%). Only approximately 60% of purchase attempts resulted in an ID check, and nearly one-half (49.5%) resulted in an underage sale (Table 1). Even after an ID check, underage sales occurred in 15.3% of visits. Electronic ID scanning occurred 22.3% of the time, and among these, only 17 (3.2%) resulted in an underage sale.

**Table 1.** Descriptive Characteristics of Tobacco Retailer Visits and Purchase Attempts, New Jersey, 2019-2022

Explanatory variable	Purchase attempts, No. (%)					
	Total	ID checked^a^	Completed sale (overall)^a^	Completed sale after ID check (n = 1569)	Electronically scanned ID (n = 2355)^a^	Completed sale after ID scanned (n = 525)
All visits	2663 (100.0)	1569 (59.0)	1318 (49.5)	240 (15.3)	525 (22.3)	17 (3.2)
Year						
2019	779 (29.3)	516 (66.2)	329 (42.2)	72 (14.0)	83 (17.6)	6 (7.2)
2021	843 (31.7)	340 (40.4)	573 (68.1)	74 (21.8)	140 (16.6)	4 (2.9)
2022	1041 (39.1)	713 (68.6)	416 (40.0)	94 (13.2)	302 (29.0)	7 (2.3)
Product type						
Cigarettes	872 (32.7)	510 (58.6)	443 (50.8)	84 (16.5)	176 (20.2)	7 (4.0)
Cigars and/or cigarillos	860 (32.3)	485 (56.5)	442 (51.4)	73 (15.1)	121 (21.9)	3 (2.5)
Electronic cigarettes	652 (24.5)	402 (61.8)	301 (46.2)	56 (13.9)	155 (23.8)	4 (2.6)
Nicotine pouches^b^	279 (10.5)	172 (61.9)	132 (47.3)	27 (15.7)	73 (26.2)	3 (4.1)
Store type						
Chain convenience	884 (33.2)	569 (64.4)	419 (47.4)	106 (18.6)	221 (27.7)	7 (3.2)
Nonchain convenience	1063 (39.9)	475 (44.8)	672 (63.3)	92 (19.4)	134 (14.7)	4 (3.0)
Gas kiosk only	178 (6.7)	98 (55.1)	92 (51.7)	14 (14.3)	18 (11.0)	2 (11.1)
Drugstore	233 (8.8)	204 (87.6)	37 (15.9)	9 (4.4)	115 (56.7)	4 (3.5)
Other^c^	305 (11.5)	223 (73.1)	98 (32.1)	19 (8.5)	37 (13.2)	0
Tobacco retailers per square mile, mean (SD) [range]	15.76 (23.68) [0.08-156.98]	NA	NA	NA	NA	NA

Abbreviations: ID, identification; NA, not applicable.

^a^ There were missing values for the following variables: ID checked (n = 4), completed sale (n = 1), and electronically scanned ID (n = 308 for cigars and/or cigarillos as this variable was added in the first week of fielding).

^b^ Product type was added in 2021.

^c^ Other store types include supermarket and/or grocery stores, vape and/or tobacco shops, dollar stores, and other types of stores.

Compared with chain convenience stores, drug stores had a higher odds of checking ID (adjusted odds ratio [aOR], 5.77; 95% CI, 1.28-26.05), whereas nonchain convenience had a lower odds (aOR, 0.49; 95% CI, 0.28-0.85) (Table 2). Compared with cigarettes, purchase attempts for nicotine pouches were associated with 0.65 (95% CI, 0.49-0.88) times the odds of checking ID. Compared with cigarettes, electronic cigarettes had 1.38 (95% CI, 1.04-1.84) times the odds of ID scan, whereas nicotine pouches had a lower odds (aOR, 0.63; 95% CI, 0.46-0.87).

**Table 2.** Unadjusted and Adjusted Associations of Retailer, Product Type Attempted, and Tobacco Retailer Availability Characteristics With ID Checks and Completed Underage Sales, New Jersey, 2019-2022^a^

Explanatory variables	ID check, yes vs no (n = 2663)^b^		ID scanned, yes vs no (n = 2355)^b^		Completed sale, yes vs no (n = 2663)^b^	
	OR (95% CI)	aOR (95% CI)	OR (95% CI)	aOR (95% CI)	OR (95% CI)	aOR (95% CI)
Store type						
Chain convenience	1 [Reference]	1 [Reference]	1 [Reference]	1 [Reference]	1 [Reference]	1 [Reference]
Nonchain convenience	0.38 (0.23-0.65)	0.49 (0.28-0.85)	0.58 (0.33-1.04)	0.53 (0.29-0.97)	2.03 (1.23-3.37)	1.24 (0.71-2.18)
Gas kiosk only	0.72 (0.37-1.39)	0.71 (0.36-1.40)	0.32 (0.08-1.32)	0.25 (0.06-0.96)	0.98 (0.49-1.95)	0.62 (0.28-1.38)
Drug store	4.18 (1.06-16.5)	5.77 (1.28-26.05)	3.18 (1.23-8.20)	3.31 (1.26-8.73)	0.24 (0.07-0.85)	0.26 (0.11-0.60)
Other	1.47 (0.87-2.82)	1.51 (0.77-2.97)	0.59 (0.30-1.17)	0.44 (0.21-0.92)	0.55 (0.29-1.03)	0.50 (0.25-0.99)
Retailer asked for ID (no vs yes)	NA	NA	NA	NA	200.71 (124.70-323.06)	265.83 (156.83-450.61)
Product type attempted for purchase						
Cigarettes	1 [Reference]	1 [Reference]	1 [Reference]	1 [Reference]	1 [Reference]	1 [Reference]
Cigars and/or cigarillos	0.94 (0.79-1.13)	0.94 (0.77-1.16)	0.91 (0.70-1.17)	0.90 (0.70-1.16)	1.00 (0.83-1.20)	0.83 (0.59-1.17)
Electronic cigarettes	1.01 (0.84-1.22)	0.97 (0.79-1.19)	1.40 (1.04-1.87)	1.38 (1.04-1.84)	0.91 (0.74-1.13)	0.79 (0.57-1.10)
Nicotine pouches^c^	0.71 (0.54-0.93)	0.65 (0.49-0.88)	0.64 (0.43-0.96)	0.63 (0.46-0.87)	1.33 (1.01-1.74)	0.96 (0.65-1.44)
Tobacco retailers per square mile	0.98 (0.97-0.99)	0.99 (0.98-1.00)	1.00 (0.99-1.00)	0.99 (0.98-1.01)	1.02 (1.01-1.03)	1.00 (0.99-1.01)

Abbreviations: aOR, adjusted odds ratio; ID, identification; OR, odds ratio.

^a^ We used generalized estimating equations to account for the nesting of visits to tobacco retailers within tobacco retailers and included a year fixed effect. Unadjusted models include each explanatory variable separately while adjusted models include all explanatory variables.

^b^ There were missing values for the following variables: ID check (n = 4), ID scanned (n = 308 for cigars and/or cigarillos as this variable was added in the first week of fielding), and completed sale (n = 1). We treated missing data as missing at random, and we thus used pairwise deletion in analyses.

^c^ Product type was added in 2021.

## Discussion

In this cross-sectional study, nearly one-half of underage tobacco purchase attempts were successful. Convenience stores and gas kiosks had the lowest rates of checking IDs and highest rates of underage sales. Retailers in areas with greater TRA may be less likely to check IDs and may need to be targeted for T21 compliance checks, especially given neighborhood racialized and socioeconomic inequities in TRA^3^ and underage sales violations.^4^ A growing number of visits implemented electronic ID scanning, and there were very few completed sales after electronic ID scanning. Electronic ID verification may help improve tobacco age of sale compliance.^5^

Our sample of retailers was limited to retailers in New Jersey (2019-2022), and results may not be generalizable. Additionally, our first wave of data collection ended just as the COVID-19 pandemic began (March 2020), and the second wave was conducted later in the pandemic (June 2021).

Despite state and federal implementation of T21, our findings suggest that retailers are not consistently checking IDs, and underage sales of tobacco products are occurring often. This study demonstrates the urgent need for continued compliance and enforcement of state and federal T21 policies. Without the full implementation of T21, the potential impact on youth and young adult tobacco use may be substantially hindered.

## Notes

Supplement 1.
eMethods.

eReferences

Supplement 2.
Data Sharing Statement
