# Supplementary material for: Identification Checks and Underage Sales of Tobacco Products in New Jersey, 2019-2022
Source: JAMA Netw Open. 2025 Jan 30;8(1):e2457319. doi: 10.1001/jamanetworkopen.2024.57319 (PMC11783190; doi:10.1001/jamanetworkopen.2024.57319)
Supplement: Supplement 1. — eMethods. eReferences [file jamanetwopen-e2457319-s001.pdf]

Supplemental Online Content

Kong AY, Hrywna M, Ackerman C, Lee JGL, Delnevo CD. Identification checks and underage sales of tobacco products in New Jersey, 2019-2022. *JAMA Netw Open*. 2025;8(1):e2457319. doi:10.1001/jamanetworkopen.2024.57319

eMethods
eReferences

This supplemental material has been provided by the authors to give readers additional information about their work.

© 2025 Kong AY et al. *JAMA Network Open.*

eMETHODS

Sample and Covert Buys

The Rutgers Health Sciences Institutional Review Board determined this study was non-human subjects research, and a full description of our protocol is described elsewhere.[1,2] In short, we created a sampling frame of all licensed tobacco retailers within a 25-mile Euclidean radius of New Brunswick, New Jersey (NJ) (N=4011). We then excluded 529 liquor stores for a total sampling frame of 3482 retailers. From both high population density municipalities stratum (n=1884) and the low population density municipalities (n=1598) stratum, we randomly sampled 50 tobacco retailers in each.

Trained research staff visited each retailer to confirm location, retailer type, and availability of tobacco product types (i.e., cigarettes, cigars, e-cigarettes, and starting in 2021, nicotine pouches) prior to purchase attempts in each wave of data collection (i.e., 2019, 2021, 2022). This resulted in a total of 14 retailers being excluded from the sample due to safety concerns, store closures, or product unavailability and resulted in a sample of 86 unique tobacco retailers in 2019. In 2021, we modified the sample to remove tobacco retailers (n=21) that sold only one type of tobacco product, which resulted in an eligible sample of 65 stores. We also added 10 vape shops within a 25-mile radius of New Brunswick, NJ and again visited stores prior to purchase attempts after which five stores were removed from the sample because of store closure or product unavailability, leaving a final sample of 60 stores and 10 vape shops. In 2022, visits prior to purchase attempts further reduced the sample of stores to 56 stores and 10 vape shops.

We recruited and trained a total of 25 covert buyers (range: 5-12 covert buyers each year of data collection) between the ages of 18 and 20 who were each assigned to make up to four purchase

© 2025 Kong AY et al. *JAMA Network Open*.

attempts with each purchase attempt being for one product type. We trained covert buyers not to lie about age and to show their legal driver's license if asked. Data collection was paused in 2020 due to the COVID-19 pandemic. This resulted in a total of 2,663 covert purchase attempts between 2019 and 2022.

Outcome Variables

Covert buyers indicated whether they were asked for ID when attempting each tobacco product purchase (1=yes, 0=no); whether their ID was electronically scanned (1=yes, 0=no), and whether the retailer completed the underage sale of each tobacco product (1=yes, 0=no). Our preliminary field assessments alerted us to the use of electronic ID verification, which we added as an indicator after the first few weeks of data collection. As such, there are 308 missing values for this variable in the first wave of data collection for cigars/cigarillos.

Explanatory Variables

We examined whether each tobacco retailer product described above was associated with outcome variables. To examine whether retailer type was associated with each outcome variable, we combined retailer type into five categories (i.e., chain convenience, non-chain convenience, gas kiosk only, drug store, other [supermarket/grocery stores, vape/tobacco shops, dollar stores, and any other types of stores]). We used ArcMap 10.7.1, to geocode the verified address of each tobacco retailer in 2019 and calculated the total number of licensed tobacco retailers per square mile within each census tract (i.e., tobacco retailer availability) for each retailer.

© 2025 Kong AY et al. *JAMA Network Open*.

Analysis

All analyses were calculated using SAS 9.4. We calculated descriptive statistics for tobacco retailer visits and purchase attempts and of ID checks and underage product sales by tobacco product type and retailer type. To test whether explanatory variables were associated with ID checks and completed purchases, we fit generalized estimating equations (GEE) with an exchangeable working correlation matrix to account for the nesting of visits to tobacco retailers within tobacco retailers and further included a year fixed effect. Unadjusted models include each explanatory variable separately while adjusted models include all explanatory variables. We treated missing data as missing at random and thus, used pairwise deletion in analyses.

© 2025 Kong AY et al. *JAMA Network Open*.

eREFERENCES

1. Hrywna M, Kong AY, Ackerman C, Hudson SV, Delnevo CD. Retailer Compliance With Tobacco 21 in New Jersey, 2019-2020. *JAMA Network Open*. 2022;5(10):e2235637-e2235637. doi:10.1001/jamanetworkopen.2022.35637

2. Hrywna M, Kong AY, Ackerman C, et al. Assessing the Effectiveness of Tobacco 21 Laws to Reduce Underage Access to Tobacco: Protocol for a Repeated Multi-Site Study. *Methods Protoc*. Mar 10 2023;6(2)doi:10.3390/mps6020027

© 2025 Kong AY et al. *JAMA Network Open*.
