# Supplementary material for: Identification Checks and Underage Sales of Tobacco Products in New Jersey, 2019-2022
Source: JAMA Netw Open. 2025 Jan 30;8(1):e2457319. doi: 10.1001/jamanetworkopen.2024.57319 (PMC11783190; doi:10.1001/jamanetworkopen.2024.57319)
Supplement: Supplement 2. — Data Sharing Statement [file jamanetwopen-e2457319-s002.pdf]

Data Sharing Statement

Kong. Identification Checks and Underage Sales of Tobacco Products in New Jersey, 2019-2022. *JAMA Netw Open*. Published January 30, 2025.
doi:10.1001/jamanetworkopen.2024.57319

Data
Data available: No

Additional Information
Explanation for why data not available: Covert buy data are available upon request to the corresponding and senior author. All other data used in the study are publicly available.
